# How Hippo Signaling Pathway Modulates Cardiovascular Development and Diseases

**DOI:** 10.1155/2018/3696914

**Published:** 2018-02-08

**Authors:** Wenyi Zhou, Mingyi Zhao

**Affiliations:** ^1^Guangdong Cardiovascular Institute, Guangdong General Hospital, Guangdong Academy of Medical Sciences, Guangzhou 510100, China; ^2^Guangzhou Medical University, The Second Affiliated Hospital of Guangzhou Medical University, Guangzhou 510000, China; ^3^Department of Pediatrics, The Third Xiangya Hospital, Central South University, Changsha 410013, China

## Abstract

Cardiovascular disease remains the leading cause of death around the globe. Cardiac deterioration is associated with irreversible cardiomyocyte loss. Understanding how the cardiovascular system develops and the pathological processes of cardiac disease will contribute to finding novel and preventive therapeutic methods. The canonical Hippo tumor suppressor pathway in mammalian cells is primarily composed of the MST1/2-SAV1-LATS1/2-MOB1-YAP/TAZ cascade. Continuing research on this pathway has identified other factors like RASSF1A, Nf2, MAP4Ks, and NDR1/2, further enriching our knowledge of the Hippo-YAP pathway. YAP, the core effecter of the Hippo pathway, may accumulate in the nucleus and initiate transcriptional activity if the pathway is inhibited. The role of Hippo signaling has been widely investigated in organ development and cancers. A heart of normal size and function which is critical for survival could not be generated without the proper regulation of the Hippo tumor suppressor pathway. Recent research has demonstrated a novel role of Hippo signaling in cardiovascular disease in the context of development, hypertrophy, angiogenesis, regeneration, apoptosis, and autophagy. In this review, we summarize the current knowledge of how Hippo signaling modulates pathological processes in cardiovascular disease and discuss potential molecular therapeutic targets.

## 1. Introduction

Heart disease continues to be the main risk of death in both developed and developing countries. Heart malformation could lead to embryonic or postnatal death, and strenuous stimulations like pressure overload and/or ischemia could cause irreversible damage. It has been shown that cardiomyocytes rapidly change from the proliferative state into hypertrophy at postnatal day 3 or 4 [1]. The regenerative ability of cardiomyocytes has been demonstrated in young human hearts [2], providing evidence that can be used toward heart regeneration therapy. However, due to the massive cell loss and the limited potential of cardiomyocyte proliferation in heart diseases, optimization of cardiac regeneration treatments remains challenging.

The Hippo signaling pathway primarily consists of the MST1/2-SAV1-LATS1/2-MOB1-YAP/TAZ cascade, known to regulate multiple organ development and diseases [3, 4]. In addition, NDR was recently included as a novel member in the cascade [5, 6]. YAP dephosphorylation leads to its inactivation, followed by cytoplasmic retention when the Hippo pathway is “switched on.” However, when the pathway is “switched off,” YAP is phosphorylated and accumulates in the nucleus, promoting cellular proliferation, metastasis, or regeneration [3, 4].

Interestingly, the Hippo pathway participates in diverse physiological and pathological processes in the heart spanning heart development, apoptosis, hypertrophy, autophagy, angiogenesis, and cardiomyocyte regeneration [7]. The purpose of this review is to summarize the current findings of the Hippo signaling cascade in cardiac development, apoptosis, hypertrophy, autophagy, angiogenesis, and cardiomyocyte regeneration. Moreover, we will explore novel therapeutic approaches in the field.

## 2. Hippo-YAP Pathway

The classical Hippo pathway was first characterized in *Drosophila*, identifying the major effectors like Hippo, Warts, Yorkie, and Mats [7]. The counterparts of these kinases in mammalian cells are MST1/2, LATS1/2, YAP/TAZ, and MOB1, respectively [7]. Here, we will discuss the most widely studied core cascade, namely, the MST1/2-SAV1-LATS1/2-MOB1-YAP/TAZ signaling pathway components. When the Hippo cassette is switched on, the activated MST1/2 (also termed STK4/3) phosphorylates LATS1/2, which in turn could cause phosphorylation of the major effectors YAP/TAZ [8]. MST1/2 is regulated by SAV1 protein, whereas MOB1 may interact with LATS1/2 [9]. Once YAP is phosphorylated, it can either be held in retention in the cytoplasm by protein 14-3-3 [8] or undergo degradation [10]. On the contrary, when the Hippo pathway is at the “off” state, YAP can no longer be phosphorylated, causing it to accumulate in the nucleus where it forms a complex together with TEAD (Transcriptional Enhancer Associated Domain) and initiates further biochemical activities [9]. Apart from the regular Hippo-YAP axis, novel kinases like NDR1/2 (STK38/STK38L), MAP4Ks, and CK1 are also included in the network [5, 6, 10]. Activated MAP4Ks may also phosphorylate both LATS1/2 and NDR1/2 [5]. While LATS phosphorylates YAP on five serine residues: S61, S109, S127, S164, and S381, NDR directly phosphorylates YAP on S127, restraining it from shuttling into the nucleus [6, 10]. Interestingly, recent research has identified another player which executes opposite effects on YAP in comparison to NDR and LATS activity [11]. Nemo-like kinase (NLK), a member of the nonclassic MAP-kinase family, phosphorylates YAP at the S128 residue. On the one hand, it deters YAP from binding with 14-3-3, and on the other hand, it reduces the phosphorylation of YAP at S127, thus promoting YAP nuclear localization [11]. What is more, the activities of LATS1/2 and NDR1/2 both rely on MOB1 [5]. The important work from Zhao et al. has provided deeper insights into the modulation of YAP protein [10]. The authors found that, after being phosphorylated on the S381 site, YAP was subjected to further phosphorylation by CK1 on S384, facilitating the degradation of YAP [10].

**Figure 1:**
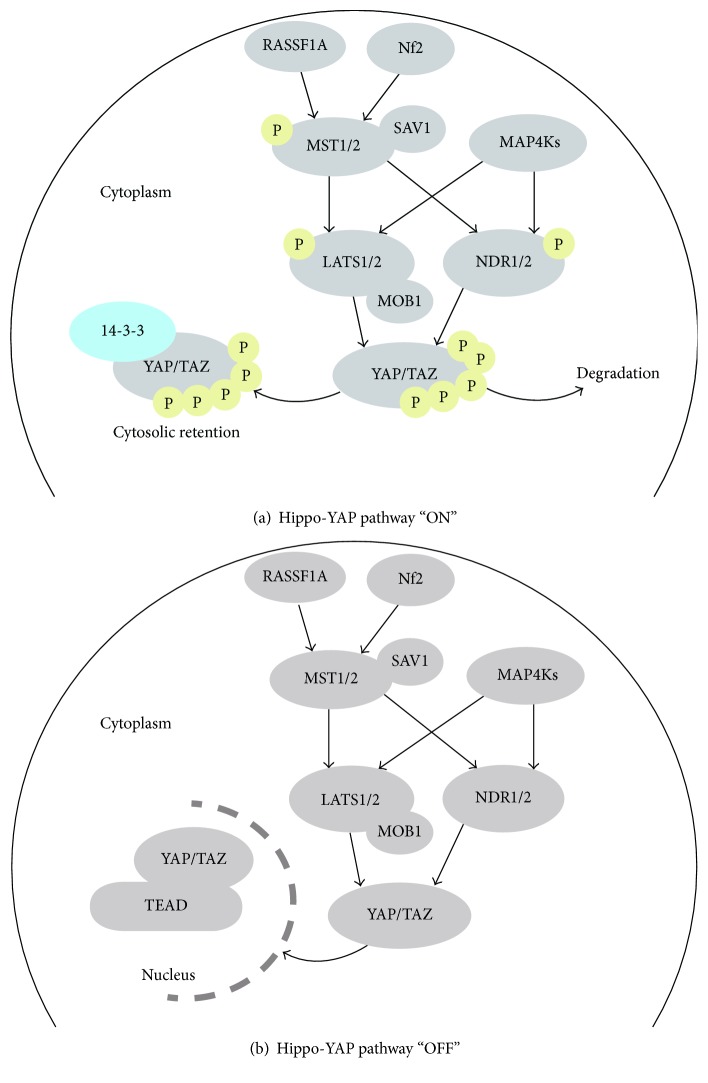
The overview of Hippo-Yap signaling pathway. (a) YAP and TAZ are phosphorylated and held in cytoplasm when the Hippo-YAP pathway is at the “ON” status. (b) Unphosphorylated YAP and TAZ accumulate in the nucleus with TEAD when the Hippo-Yap pathway is at the “OFF” status.

There are two additional renowned molecules positively regulating MST1/2. One of them is RASSF1 (Ras association domain family 1), a tumor suppressor member protein [12]. RASSF1A may keep MST1/2 at its phosphorylated state and prevent dephosphorylation by binding to MST1/2 [12]. The other molecule is Nf2 (neurofibromin2), a tumor suppressor and a proapoptosis kinase, which positively regulates the Hippo signaling through activation of MST1 [13]. The summary of the Hippo-Yap signaling pathway is shown in Figure 1.

The Hippo-YAP pathway primarily functions in cell proliferation and apoptosis, controlling the organ size [4]. Cell polarity, cell contact, other mechanical forces, and soluble factors were identified as key Hippo signaling regulators [3, 4]. To our knowledge, whether YAP could exert biochemical functions depends on its location. Intriguingly, cell density could also affect the YAP localization and vice versa [8]. When YAP is upregulated, cell growth may be stimulated without the constraints of cell-contact inhibition [8]. Except for numerous upstream factors regulating the Hippo pathway, the cascade itself has a negative feedback loop. YAP coupled with TEAD in the nucleus may augment the expression of Nf2, LAST2, and MST1, which together may ensure negative control on YAP [4].

## 3. Role of the Hippo Tumor Suppressor Pathway in Cardiovascular Disease

The summary of how the Hippo-YAP pathway participates in cardiovascular development, hypertrophy, apoptosis, autophagy, angiogenesis, and regeneration is listed in Table 1.

### 3.1. The Hippo-YAP Signaling in Cardiovascular Development

Numerous studies have documented the essential role of the Hippo tumor suppressor pathway in organ development and tumorigenesis. In 2011, Xin et al. showed that deletion of YAP in mice containing the Nkx2.5-Cre resulted in reduced cardiomyocyte proliferative ability and eventually led to embryonic death at stage 10.5. On the other hand, cardiomyocyte numbers in the newborn mice were significantly increased when YAP expression was increased with adenovirus expressing YAPS112A [14]. In addition, the proproliferation function of YAP was achieved via activation of the insulin-like growth factor pathway [14]. Heallen et al. reported that cardiac-specific knockout of SAV1 inactivates the Hippo signaling, evident by decreased phosphorylated YAP level but not total YAP, leading to an enlarged heart without alterations in cell size. Similar results were found in MST1/2 and LATS2 knockout mice [15]. To deepen insights into the underlying mechanisms, their group also reported that the Hippo pathway exerts its antigrowth effects through suppression of Wnt signaling [15]. While ventricular septal defect (VSD) was observed in some of the SAV1 mutant hearts [15], VSD also occurred in cardiac/vascular smooth muscle cell- (SMC-) specific YAP ablation mice, which may harbor other severe vascular malformations like dysplastic arterial wall, hypogenetic brachiocephalic artery, and retroesophageal right subclavian artery [16]. These abnormalities may ultimately lead to perinatal fatality [16]. To further elucidate on the role of Hippo signaling in coronary development, Singh et al. induced epicardial-specific deletion of YAP and TAZ with Sema3dGFPCre+/−. The epicardium is one of the important sources of coronary vasculogenesis [17]. Results showed that YAP/TAZ-null mice embryos had attenuated differentiation of the epicardial cell into coronary endothelial cells which caused embryonic lethality between stages E11.5 and E12.5 [17]. These data emphasize the significant role of the Hippo pathway in normal cardiovascular development.

### 3.2. The Hippo-YAP Signaling in Cardiomyocyte Hypertrophy and Apoptosis

Cardiomyocytes and fibroblasts are two critical components of the heart. In response to hypertension or other pressure overload diseases, cardiac hypertrophy occurs, resulting in enlargement of cell size, enrichment of cell number, or both. However, given that adult cardiomyocytes have limited proliferation ability, cardiomyocyte hypertrophy rather than hyperplasia have more commonly been observed. To investigate the possible role of Hippo signaling in cardiomyocyte hypertrophy and apoptosis, we discuss its key components separately.

To start with MST1, Lin et al. have summarized that altered expression levels of MST1 could not affect the size of cardiomyocytes but an upregulated MST1 level increased cardiomyocyte apoptosis [18]. Other researcher groups have reported that the RASSF1A/MST1 pathway exerts rather different results in cardiomyocyte and fibroblast. That is, in a setting of pressure overload, activated RASSF1A/MST1 may lead to cardiomyocyte apoptosis, while suppressing the proliferation ability of fibroblast leading to reduced cardiac hypertrophy [19]. This interesting finding may indicate that specific inhibition of the RASSF1A/MST1 pathway in cardiomyocytes rather than fibroblasts could be a novel therapeutic target [19]. Based on the evidence that MST2 knockout mice showed attenuated hypertrophy while MST2 overexpression lead to increased hypertrophy, researchers concluded that MST2 took part in cardiac hypertrophy [20]. However, such effect was exerted through the Raf1/ERK1/2 pathway but not by alteration of YAP [20]. When it comes to LATS1/2, Matsui et al. reported that LATS2 contributed to MST1-mediated apoptosis and antihypertrophy in the heart but upregulation of LATS2 alone did not affect cardiac apoptosis at baseline [21]. Moreover, LATS2 reduced cardiomyocyte size in vitro in a dose-dependent manner, and it negatively modulated cardiac hypertrophy in response to pressure overload [21]. Consistently, silencing LATS1 with siRNA enhanced the paracrine secretion of cardiac fibroblasts when under mechanical stress stimulation, leading to cardiac hypertrophy [22]. As for YAP, the major effector of the Hippo pathway, recent data has shown that YAP activation plays an essential role in cell proliferation of both fetal and postnatal heart while not affecting cardiomyocyte size in both physiological and pathological settings [23]. Lin et al. also found that in the context of myocardial infarction (MI), YAP activation exerted its cardioprotective function via stimulation of cell proliferation without causing hypertrophy [24]. However, another team which also investigated the role of YAP in cardiomyocytes found that YAP also induced cardiac hypertrophy in addition to its antiapoptotic and proproliferative role [25]. While YAP negatively controls cardiomyocyte apoptosis through activation of Akt [25], it could also encourage compensatory cardiomyocyte hypertrophy via upregulation of miR-206 [26], both favoring cell survival.

In summary, the Hippo pathway which primarily functions through the YAP effector protein, mediates cardiac hypertrophy and apoptosis, although it seems that whether YAP could induce hypertrophy depends on different backgrounds. However, noteworthily, the core components of the Hippo cascade might cause hypertrophy or apoptosis via other pathways without translocating the YAP protein.

### 3.3. The Hippo-YAP Signaling in Angiogenesis

Angiogenesis, a process that produces neovessels, is important in the context of cardiac ischemia. Given that endothelial cells (ECs) play a major part in angiogenesis and the Hippo-YAP pathway participates in EC survival, proliferation, and migration [27], abundant evidence has supported the idea that Hippo signaling could tune the production of new blood vessels. Choi et al. demonstrated that the tube formation or sprouting ability of ECs was noticeably diminished after was YAP knocked down by short interfering RNA [28]. As previously shown, Zhao et al. found that YAP localization was mediated by cell-cell contact at least partially driven via the Hippo pathway [8]. Interestingly, Choi et al. discovered that phosphorylation of YAP in ECs was regulated by VE-cadherin through Akt but not mediated by LATS1/2 [28]. Moreover, upregulation of YAP could induce robust angiogenesis by transcriptional modulation of angiopoietin-2 [28]. Similarly, Marti et al. revealed another transcriptional target of YAP in cholangiocarcinoma which enhances neovascularization, that is, microfibrillar-associated protein 5 (MFAP5) [29]. Apart from YAP, Dai et al. helped define that Angiomotin is an alternative target of LATS1/2. Angiomotin phosphorylation on Serine175 mediated by LATS1/2 negatively regulates angiogenesis in zebrafish embryos [30]. Furthermore, Yuan et al. demonstrated that palmitic acid interfered with the Hippo pathway by blocking YAP from shuttling into the nucleus leading to attenuated angiogenesis. This process was mainly driven by palmitic acid damage on mitochondria [31]. In conclusion, these data support the proangiogenesis role of YAP. Surprisingly, the activated LATS1/2 could negatively modulate angiogenesis via regulation of YAP and other targets.

### 3.4. The Hippo-YAP Signaling in Heart Regeneration

Myocardial infarction (MI) is a devastating disease around the world because it causes irreversible cell loss in the heart. The limited proliferation capacity of the adult cardiomyocytes makes the management of MI rather challenging. In recent years, therapies aiming at promoting the regeneration ability of the heart have attracted much spotlight. Among these therapies, harnessing the Hippo pathway might be beneficial. Xin et al. investigated the role of YAP in the context of MI [32]. When neonatal hearts were subjected to left anterior descending coronary artery ligation, mice in the cardiac-specific YAP knockout group showed broader infarcted area with reduced functioning cardiomyocytes. Furthermore, the proproliferation and prosurvival function of YAP was observed in a postnatal MI model [32]. Lin et al. used an adeno-associated virus serotype 9 (AAV9) to specifically upregulate YAP in an in vivo heart model. The authors successfully identified a direct target of YAP, the Pik3cb, which regulates the Hippo signaling through the PI3K-Akt pathway [33]. By increasing the expression level of YAP, the cardiomyocyte number and heart function were restored after MI at least partially through Pik3cb [33]. Moreover, Tian et al. provided novel insights into microRNA-based heart regeneration therapy [34]. They identified a cluster of microRNA, miR302–367, which drives the cardiomyocyte to reenter the cell cycle, thus inducing cardiac proliferation after MI, fractionally due to inhibition of the Hippo pathway [34]. However, constitutive expression of the miRNA was not beneficial. To overcome this problem, Tian et al. found that utilization of miR302–367 could achieve the same desired outcome but with minimum side effects [34]. Intriguingly, Tao et al. described another transcription factor, the paired-like homeodomain transcription factor 2 (Pitx2), but with subsidiary function in the regulation of YAP [35]. Subsequent studies showed that Pitx2-deficient mice failed to regrow. Furthermore, Pitx2-overexpressing in mice after apex dissection showed successful functional recovery in the heart. This study indicates a heart-regenerating role of Pitx2, probably achieved through YAP interaction to promote cardiomyocyte entry into the S-phase [35]. Together, these data underline the indispensable role of the Hippo-YAP pathway in heart regeneration.

### 3.5. The Hippo-YAP Signaling in Cardiomyocyte Autophagy

The term autophagy was first described by Christian de Duve in 1963, referring to a self-protective process in which damaged organelles and defective proteins were decomposed and recycled [36]. There are grossly three kinds of autophagy, macroautophagy, microautophagy, and chaperone-mediated autophagy [37]. However, here we will primarily discuss macroautophagy (referred to as autophagy in this article) since it is the most studied pathway. There are several phases in the autophagy process. Stimulation like starvation (especially depletion of amino acids) could induce the initial step of autophagy [37], through the inactivation of mTORC1 (i.e., the mammalian target of rapamycin complex 1), which could in turn positively regulate the ULK1 complex [38]. On the other hand, when stimulated by insulin or other growth factors, class I PI3K-AKT could inhibit autophagy via two separate pathways: activation of mTORC1 and suppression of Beclin1-VPS34 complex [38]. After initiation, products destined for degradation are recruited, and the isolated membrane elongates, closes, and eventually fuses with lysosomes [36]. The membrane of autophagosomes comes from endoplasmic reticulum, Golgi complex, mitochondria, and plasma membrane. The formation of autophagosomes is primarily governed by ATG proteins, among which the Atg12-Atg5-Atg16L1 complex and LC3I-PE complex (LC3II) are equally essential [36–38]. More details regarding ATG proteins are described in Choi et al.'s outstanding review [38]. In physiological conditions, autophagy maintains cellular homeostasis, controls the quality of mitochondria, and contributes to organ development by degrading nonfunctional organelles and proteins. This checkpoint control assures that degradation is not simply a waste. Autophagy can process cellular components into amino acids, lipids, and sugar constituents, which later could be utilized in protein synthesis or production of glycogen or act as a direct supply for ATP [38, 39]. The significance of autophagy in cardiac development was confirmed by evidence that specific ATG5 knockout in the heart could ultimately lead to malformation and dysfunction of the heart, resulting from loss of autophagy [40]. Furthermore, ATG5 upregulation increased lifespan probably due to enhanced autophagy [41].

MST1, a proapoptotic effector in cardiomyocytes [7], concomitantly modulated autophagy in response to stress [42]. Apart from its central role in the Hippo-YAP pathway, MST1 also phosphorylates Beclin1 on the Thr108 residue in the BH3 domain, disturbing the Beclin1-PI3K class III complex, thus reducing autophagy flux [42]. Of note, the Beclin1 phosphorylation strengthens the stability of the Beclin1-Bcl-2 complex, thus promoting apoptosis [42]. Current reports indicate that MST1 may be involved in the pathogenesis of atherosclerosis progression [43], diabetic coronary microvascular dysfunction [44], and diabetic cardiomyopathy [45] by increasing apoptosis and suppressing autophagy. The indispensable role of MST1 in cardiac autophagy was demonstrated by Sun et al.'s research [46–50]. In mice subjected to diabetic cardiomyopathy or myocardial infarction (MI) induced-surgery, Melatonin promoted heart function by enhancing autophagy and weakening apoptosis, as indicated by the elevated expression levels of Beclin1, Atg5, LC3II, and decreased p62. Moreover, this effect was observed by inhibition of MST1 phosphorylation, and it could be abolished in the MST1 double knockout mice [46, 49]. Similarly, the protective effect of oncostatin M (OSM) in MI mice model was subverted by knockout of MST1 [47]. Furthermore, Sirt3, a downstream regulator of MST1, was found to be a key modulator of autophagic flux in cardiomyocytes under the treatment of Polydatin in an MI model [50]. However, a recent study has identified LC3 as a novel target of MST1/2, and phosphorylation of LC3 on its Thr50 residue by MST1/2 can promote the fusion step of autophagy [51].

NDR1, another proapoptotic protein, plays an essential role in autophagy [5, 52] by resembling LATS1/2. NDR1 functions as the positive upstream phosphorylation regulator of YAP [5]. NDR1 is required in the early stage of autophagy through interactions with Beclin1 [52]. Overactivation of NDR1 may cause apoptosis even though it was in the case of autophagy induction [52]. To investigate mechanisms balancing between apoptosis and autophagy, Joffre et al. [52] successfully revealed that RalB restrained the activity of NDR1 by preventing hyperactivation.

Moreover, it has been shown that YAP also participates in the autophagy process, playing a protective role in breast cancer [53]. However, its role in cardiomyocytes is worthy of further elucidation.

## 4. Possible Therapeutic Insights

Homeostasis has vital importance in maintaining the normal physical function of the cardiovascular system, thus Hippo signaling, and autophagy can play an important role. Discovering that autophagy has a cardioprotective role in MI, Kanamori et al. suggested a therapeutic role in treating MI [54]. Recent studies have confirmed this theory and demonstrated that Melatonin, Luteolin, and OSM can enhance the autophagic flux and suppress apoptosis by subverting phosphorylation of MST1, ultimately attenuating cardiac dysfunction after MI [46–48]. Moreover, Melatonin can also exert a similar effect on diabetic cardiomyopathy [49], while Polydatin demonstrated to beneficially elevate autophagic flux and limit cellular apoptosis in the context of MI [50]. Fan et al. described an interesting phenomenon. AS-1 (hydrocinnamoyl-L-valyl pyrrolidine), a TIR/BB-loop mimetic, can protect cardiomyocytes from hypertrophy in the context of pressure overload by partially increasing phosphorylation of LATS1 levels [22]. Moreover, acetylation of VGLL4, a tumor suppressor, can stimulate the combination activity of YAP and TEAD, providing an interesting insight of their dual role in heart regeneration [55].

In conclusion, our current understanding of the Hippo pathway and its role in the cardiac field remains insufficient. Furthermore, the Hippo pathway might have a different role in different types of cells. Further knowledge about its underlying mechanisms may help identify novel therapeutic targets.

## Figures and Tables

**Table 1:** Role of the Hippo tumor suppressor pathway in cardiovascular disease.

	Effecter	Methods	Outcomes	Ref.
Cardiovascular development	MST1/2	Cardiac-specific knockout of MST1/2	Enlarged hearts; without alteration of cell size	[15]
	LATS2	Cardiac-specific knockout of LATS2	Enlarged hearts; without alteration of cell size	[15]
	SAV1	Cardiac-specific knockout of SAV1	Enlarged ventricular chambers; thickened ventricular walls; without alteration of cell size	[15]
	YAP	Deplete YAP with Nkx2.5-Cre	Reduced cardiomyocyte proliferative ability; embryonic death at embryonic stage 10.5	[14]
		Overexpress YAP with adenovirus	Cardiomyocytes number increased significantly in newborn mice	[14]
		Cardiac/vascular smooth muscle cell-specific ablation of YAP	Vascular malformations like ventricular septal defect etc.; might result in perinatal fatality	[16]
		Epicardial-specific deletion of YAP/TAZ with Sema3dGFPCre+/−	Attenuated differentiation of the epicardial cell into coronary endothelial cells; embryonic death between E11.5 and E12.5	[17]

Cardiomyocyte hypertrophy and apoptosis	RASSF1A	Generated RASSF1A transgenic (TG)/(L308P) RASSF1A TG mice with adenoviral system; subjected them to pressure overload	Increased MST1 phosphorylation; promotes cardiomyocyte apoptosis; reduced the proliferation ability of fibroblast and cardiac hypertrophy	[19]
	MST1	Upregulation of MST1	Enhanced cardiomyocyte apoptosis	[18]
	MST2	MST2 knockout	Attenuated cardiac hypertrophy	[20]
		MST2 overexpression	Increased cardiac hypertrophy	[20]
	LATS1	Mutation of LATS1 using siRNA	Encouraged cardiac hypertrophy	[22]
	LATS2	Transduced Ad-LATS2 or Ad-LacZ into cultured myocytes; generated LATS2 and DN-LATS2 TG mice using the *α*-myosin heavy chain promoter	Dose dependently increased apoptosis and reduced cardiac myocyte size in vitro; negatively regulated ventricular chamber size in vivo	[21]

Cardiomyocyte hypertrophy and apoptosis	YAP	Cardiac-specific activation of YAP using adenoassociated virus subtype 9 (AAV9) after MI	Improved cardiac function without causing hypertrophy; enhanced survival	[24]
		Cardiac-specific inactivation of YAP1 using *α*-MHC Cre recombinase transgenic mice; transduced cardiomyocytes with YAP1 or LacZ adenovirus	Caused increased cardiomyocyte apoptosis in YAP(−/−) at baseline; YAP expression induced cardiomyocyte hypertrophy	[25]

Angiogenesis	LATS1/2	Coinjection of mRNAs encoding Angiomotin p130 and mRNAs encoding LATS2	Induced angiogenesis defects in zebrafish embryos	[30]
	YAP	Knock-down of YAP by siRNA	Significantly reduced the tube formation or sprouting ability of endothelial cells	[28]
		Upregulation of YAP	Induce robust angiogenesis	[28]

Heart regeneration	YAP	Cardiac-specific YAP knockout in MI mice	The infract area was broader and cardiomyocytes were less robust	[32]
		Cardiac-specific upregulation of YAP in MI mice with adenoassociated virus serotype 9	Rescued the cardiomyocyte number and cardiac function	[33]
		Compared Pitx2-deficient mice and Pitx2-overexpressing mice when subjected to apex dissection	Pitx2-deficient mice fail to repair while Pitx2-overexpressing mice showed functional recovery	[35]

Cardiomyocyte autophagy	MST1/2	Inhibition of MST1 phosphorylation with Melatonin, oncostatin M etc.	Promoted cardiac function, enhanced autophagy, and weakened apoptosis	[46–50]
		Phosphorylation of LC3 by MST1/2	Promoted the fusion step of autophagy	[51]
	NDR1	Interact with Beclin1	Function in the early stage of autophagy	[52]
